# Atraumatic elbow avascular necrosis in the adult is rare, multifocal, and associated with systemic corticosteroid use

**DOI:** 10.1016/j.jseint.2024.10.008

**Published:** 2024-11-14

**Authors:** Daniel Chiou, Bailey Mooney, Andrew R. Jensen

**Affiliations:** a Department of Orthopaedic Surgery, University of California Los Angeles, Los Angeles, CA, USA; b David Geffen School of Medicine, University of California Los Angeles, Los Angeles, CA, USA

**Keywords:** Elbow, Avascular necrosis, Radiograph, Magnetic resonance imaging, Steroids, Elbow arthroscopy

## Abstract

**Background:**

Atraumatic avascular necrosis (AVN) of the elbow is a rare diagnosis with little literature describing features of this pathology. The purpose of this study is to investigate atraumatic elbow AVN in adults, with a focus on the anatomic distribution of AVN within skeletally mature elbows.

**Methods:**

A retrospective chart review was conducted on six patients who were identified via term searches of elbow magnetic resonance imaging (MRI) done at the authors’ institution that also had appropriate diagnoses. Terms included “necrosis”, “AVN”, and “avascular”. Demographic data were collected, including age of diagnosis, sex, associated comorbidities, use of steroids, use of chemotherapeutic agents, alcohol consumption, smoking status, and associated joint involvement. Clinical information regarding presentation and treatment course were also gathered. Both plain film and MRI were evaluated for identification of anatomic involvement of disease and staging.

**Results:**

Six patients were included in the study: three men and three women with a mean age of 26.5 years (17-46) at time of diagnosis. All patients presented with elbow pain and one patient presented additionally with loss of full range of motion. Four of the six patients had a prior cancer diagnosis (T-cell acute lymphoblastic leukemia x2, follicular lymphoma, acute myeloblastic leukemia) that led to chemotherapy exposure, and two of them had additional steroid therapy. Another two had autoimmune diseases (systemic lupus erythematous and dermatomyositis) that required high dose steroid therapy. At time of initial imaging, the capitellum was involved in 8 of 9 elbows, the trochlea in 8 of 9 elbows, the radial head in 4 of 9 elbows, the proximal ulna in 2 of 9 elbows, and the olecranon in 1 of 9 elbows. Only one elbow had additional sites of the elbow affected at future follow-ups. One patient presented with AVN of the capitellum, trochlea, and ulnar neck, and two years later had signs of olecranon osteonecrosis on MRI. Two patients underwent operative treatment with resolution of symptoms.

**Conclusion:**

This study describes the anatomic incidence of AVN of the elbow. Most involved are the capitellum and trochlea, with involvement in the radial head, proximal ulna, and olecranon also being observed. This information can be used to help orthopedic surgeons in their diagnosis and clinical decision making for affected patients.

Atraumatic avascular necrosis (AVN), or osteonecrosis, is characterized by death of bone cellular components secondary to interruption of subchondral blood supply.^6^^,^^10^ Clinically, this results in progressive pain and limited function of affected joints. Some have posited that vessel obstruction, osteocyte metabolism alteration and genetic factors may also play a role.^2^ Risk factors such as repetitive microtrauma, systemic corticosteroid use, exposure to chemotherapy, alcohol consumption, systemic lupus erythematous (SLE), and underlying metabolic genetic conditions have also been implicated as causative factors for AVN.^14^ AVN typically affects the epiphyses of long bones such as the femoral head and the humeral head, which are the two most common locations.^8^ Other common sites include the talus, carpal bones, and the mandible.^10^^,^^14^

AVN of the elbow is a relatively rare entity, with few studies describing its natural history. Of these few studies, most focus on posttraumatic or pediatric elbow AVN.^1^^,^^5^^,^^8^ Few studies have characterized elbow AVN disease progression in the adult population.^1^^,^^9^ Flouzat-Lachaniette et al presented a cohort of 50 elbows that were followed for 17 years nonoperatively, showing the development of pain and disease progression, while Le et al presented nine patients, three of whom underwent operative treatment with an average of three-year follow-up. Unlike AVN of other joints such as the hip and the shoulder, elbow AVN has been reported to affect various bones within the joint, including the distal humerus, the proximal radius, and the proximal ulna.^1^^,^^8^^,^^9^^,^^13^ The relative distribution of AVN in these locations, comprising the totality of “elbow” AVN, is currently unknown but would be important information for better understanding the etiology of atraumatic adult elbow AVN, its natural history, and hence possible remedies.

The purpose of this study was to investigate atraumatic elbow AVN in adults, with a focus on the anatomic distribution of AVN within the skeletally mature elbow. We hypothesized that the majority of elbow AVN cases would affect the capitellum predominantly, but that there would be substantial variation in AVN locations about the elbow.

## Methods

This study was a retrospective review of all orthopedic patients seen at one academic institution between the years 2013 and 2023. After institutional review board approval, an analysis was conducted from this patient population of those who had a diagnosis of “elbow pain”. Clinical diagnoses from patient records were queried using International Classification of Diseases codes. From this cohort, patients were filtered using term-searches of “necrosis”, “AVN”, and “avascular” from elbow magnetic resonance imaging (MRI) imaging reports conducted at the authors’ institution from March 19, 2013, to April 10, 2023. Six individual patients with nine affected elbows were identified. Outpatient clinic notes, inpatient hospital notes, and radiographs were reviewed for each of these patients. Demographic data were collected including age at time of diagnosis, sex, associated comorbidities, use of steroids, use of chemotherapeutic agents, alcohol consumption, smoking status, and associated nonelbow joint involvement.

Clinical course data were also collected including presenting symptoms, length of time between first visit and diagnosis, length of time between diagnosis and intervention, and description of the intervention. All elbows were treated with activity modification, physical therapy, and analgesics. Two of the nine elbows underwent further operative treatment consisting of core decompression with arthroscopic débridement, with one planned for the future.

**Figure 1:**
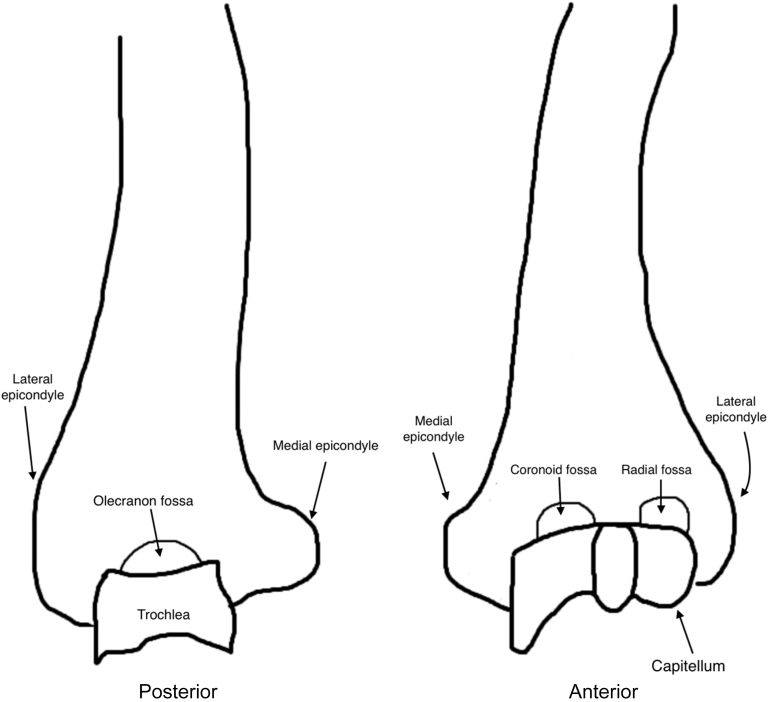
Anatomic locations of the distal humerus.

**Figure 2:**
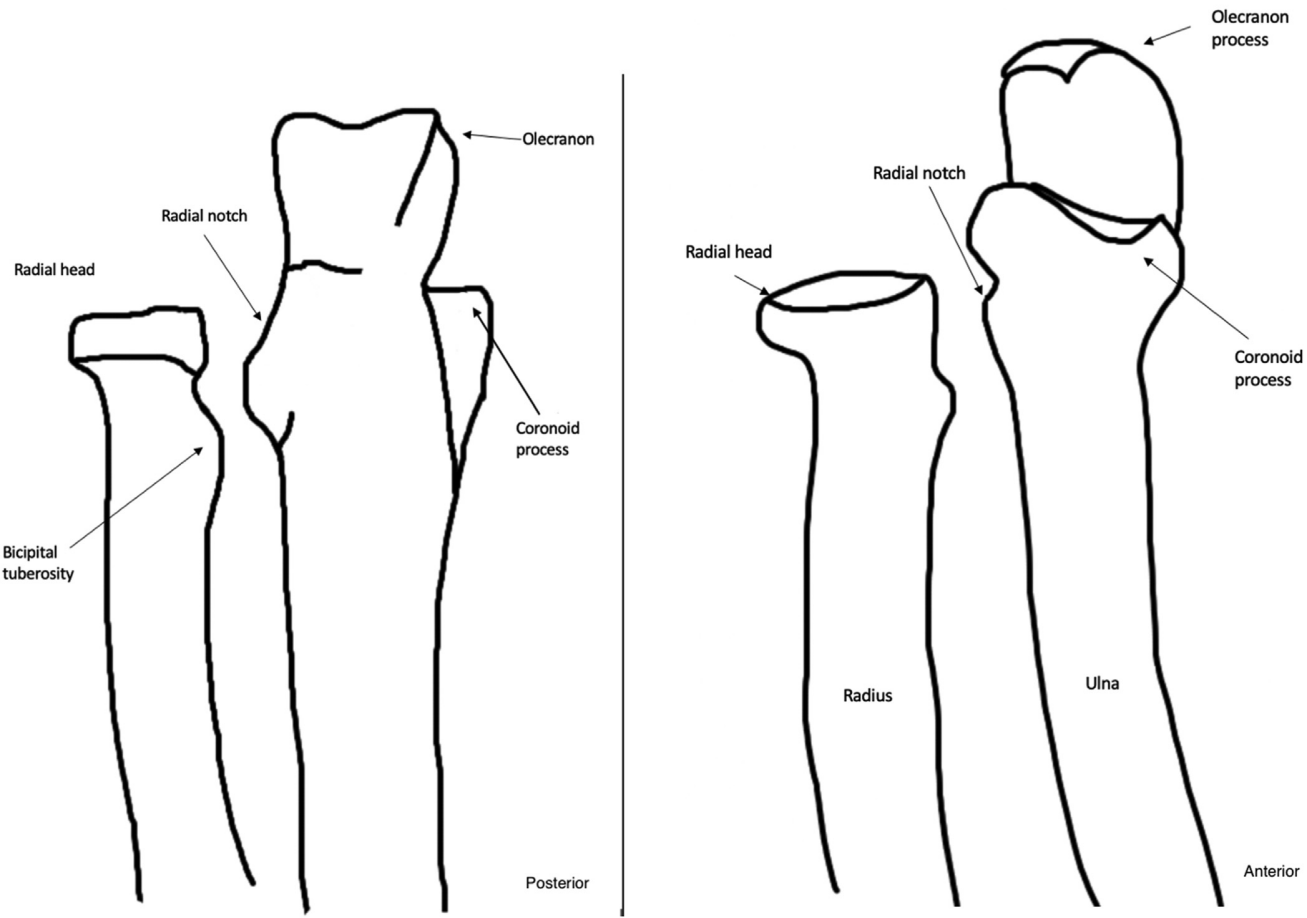
Anatomic locations of the proximal radius and ulna.

Magnetic resonance images were assessed for the anatomic distribution of the patient’s elbow AVN at time of diagnosis. Figs. 1 and 2 provide the anatomic definitions utilized for this study. All patients underwent imaging at further time points allowing for evaluation of the evolution of the disease location. Extent of disease was graded on a scale previously utilized by Floutzat-Lachaniette et al for adult elbow AVN (Table I).^1^ Both plain film and MRI with and without contrast were used when available. All patients had both plain films and MRI of their elbow at least once.

**Table I:** Osteonecrosis staging.

Stage	Criteria for staging
I	Abnormal MRI, normal radiograph
II	Abnormal radiograph, but no crescent line
III	Abnormal radiograph with a crescent sign
IV	Collapse of the articular surface without joint space narrowing
V	Joint space narrowing

*MRI*, magnetic resonance imaging.

## Results

### Clinical and demographic data

From 2013 to 2023, there were 6 patients with a diagnosis of elbow AVN: three men and three women with a mean age of 26.5 years (17-46 years) at time of diagnosis. All patients presented with elbow pain and one patient presented additionally with loss of full range of motion (ROM). Four of the six patients had a prior cancer diagnosis (Table II) that was treated with chemotherapy and high-dose systemic corticosteroids. The other two patients had autoimmune diseases (SLE and dermatomyositis) that also required high-dose systemic corticosteroid therapy. Four of the patients consumed alcohol, two on an occasional basis, and two on a weekly basis. None of the patients were smokers.

**Table II:** Demographic information.

Patient ID	Age	Sex	Past medical history	Alcohol use	Prior AVN diagnoses	Elbow involvement	Presenting symptom	Relevant medications
1	18	F	T-cell acute lymphoblastic leukemia	N	Bilateral distal femurs, bilateral proximal tibia	Left	Pain	Vincristine, cytarabine, methotrexate, doxorubicin, danorubicin, prednisone, asparginase, mercaptopurine, thioguanine, cyclophosphamide AALL0232
2	34	F	Systemic lupus erythematosus	Y	Bilateral hips	Left	Loss of range of motion, pain	Varying doses of prednisone
3	46	M	Follicular lymphoma	Y	Right hip	Left	Pain	s/p allogenic stem cell transplant;
4	22	M	Acute myeloblastic leukemia	Y	Bilateral femoral heads, right distal tibia, right talus, right navicular, right cuneiforms	Bilateral	Pain	cytarabine, daunorubicin, etoposide; received BMT cytarabine, fludarabine, and idarubicin with lymphocyte infusion; daunorubicin, and cytarabine; got azacitadine; had steroid injection throughout chemo regimen.
5	17	F	Dermatomyositis	N	Bilateral knees, bilateral ankles	Bilateral	Pain	high dose steroid; methotrexate, mycophenolate, gamma globulin
6	32	M	T-cell acute lymphoblastic leukemia	Y	Bilateral hips, bilateral shoulders	Bilateral	Pain	Vincristine, danorubicin; prednisone + dexamethasone

*AVN*, atraumatic avascular necrosis; *BMT*, bone marrow transplant.

All patients had prior diagnoses of AVN of at least one other joint at the time of elbow AVN diagnosis. Two patients had multifocal osteonecrosis (localized at other joints). Of the other sites affected by AVN, the hip was most common in four of the six patients (bilateral hip involvement in three of these four). Other sites included the knee, shoulder, ankle, and foot bones (navicular and cuneiforms).

Time from first visit until diagnosis was a mean of 35 days (7-91 days) for the five patients who had their initial diagnosis at our institution’s facilities. For the five of the six patients who received further treatment for their elbow AVN (bisphosphonate therapy, physical therapy, arthroscopic decompression, and core decompression), time between diagnosis and initiation of therapeutic intervention was a mean of 40 days (0-124 days).

### Imaging data

**Figure 3:**
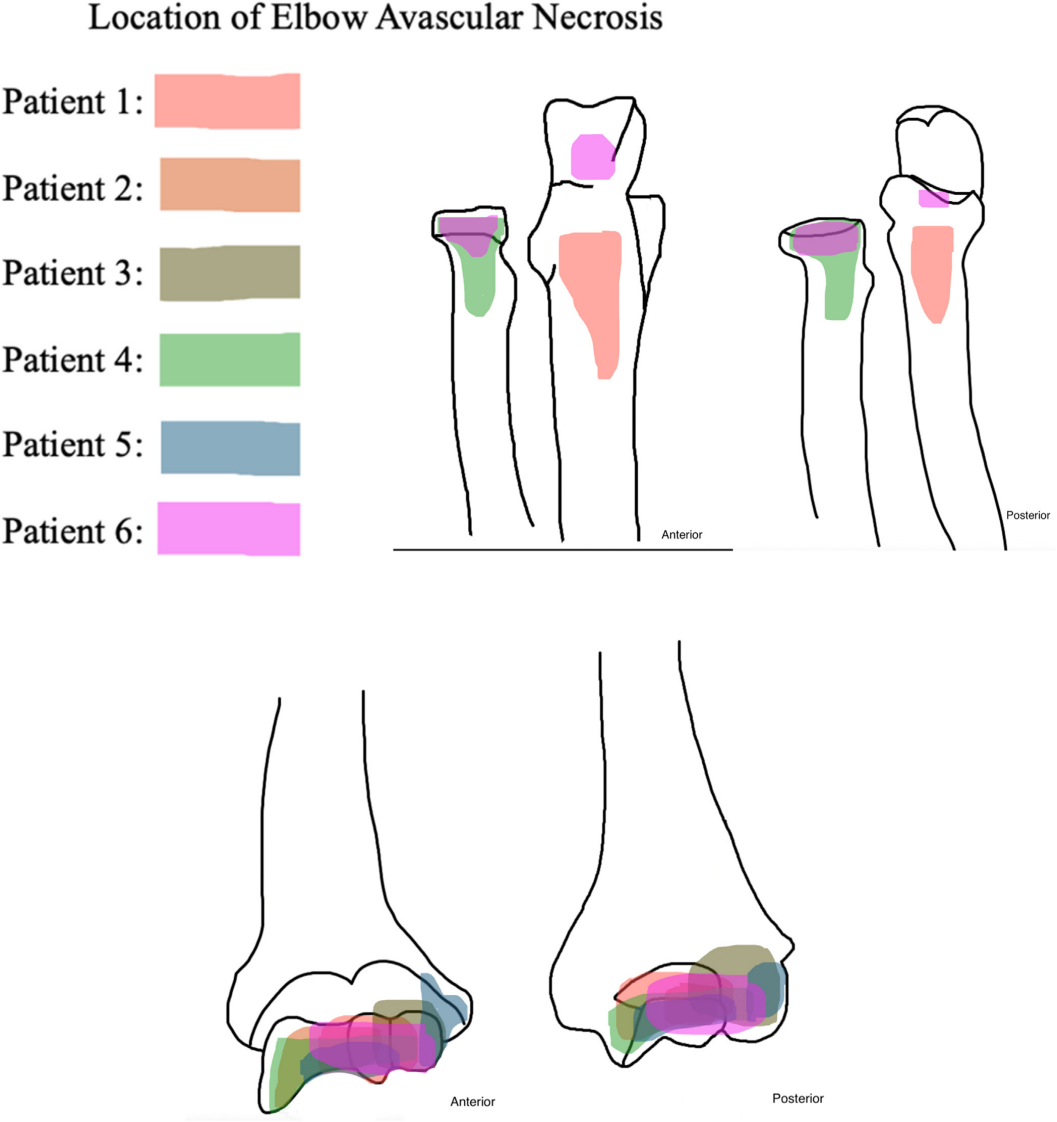
Location of AVN for each of the patients in the cohort. *AVN*, atraumatic avascular necrosis.

Figs. 1 and 2 define the anatomic locations of the elbow. At time of initial MRI, the capitellum was involved in 8 of 9 elbows, the trochlea in 8 of 9 elbows, the radial head in 4 of 9 elbows, the proximal ulna in 2 of 9 elbows, and the olecranon in 1 of 9 elbows. Only one elbow had a new area of the elbow affected at future follow-up which was not present on initial imaging; patient 1 initially presented with AVN of the capitellum, trochlea, and ulnar metaphysis alone but two years later this patient had involvement of the olecranon as well. Fig. 3 displays the locations of AVN for all six patients in the cohort.

Using the scale outlined in Table I, initial imaging for all patients was graded on severity of their osteonecrosis. At the initial visit two elbows were stage I, one elbow was stage II, two elbows were stage III, three elbows were stage IV, and one elbow was stage V. At a mean follow-up of 1.7 years, two elbows were stage I, one elbow was stage II, one elbow was stage III, and five elbows were stage IV (Table III).

**Table III:** Avascular necrosis location and staging of cohort.

Patient ID	AVN location at diagnosis	Staging at diagnosis	Additional sites of AVN after diagnosis	Staging at most recent follow-up
1	Left capitellum, trochlea, ulnar neck	IV	Left olecranon	IV
2	Left capitellum, trochlea	V	None	V
3	Left capitellum, trochlea	IV	None	IV
4	Left capitellum, trochlea, and radial head	III	None	IV
	Right trochlea	I	None	II
5	Left capitellum, trochlea	IV	None	IV
	Right capitellum, trochlea, and radial head	III	None	III
6	Left capitellum, trochlea, proximal radius, proximal ulna	I	None	I
	Right capitellum, trochlea, radial head, olecranon	II	None	I

*AVN*, atraumatic avascular necrosis.

### Treatment outcomes

Patient 2 and Patient 6 both underwent arthroscopic débridement and core decompression. Patient 2 presented with pain and stiffness of the left elbow and was found to have stage V elbow AVN disease. This patient underwent an arthroscopic synovectomy, débridement, loose body removal, and core decompression. At the most recent follow-up appointment, which was four months postprocedure, there was resolution of the pain and mild improvement in ROM (preoperative ROM: 30-130, postoperative ROM: 20-120).

Patient 6 presented after developing right elbow pain during a gym session performing bicep curls and was found to have stage II disease. Arthroscopic débridement and core decompression in the right elbow was performed. At the most recent follow-up (9 months postoperatively), there was improvement of pain symptoms, return to full ROM, and ability to lift up to 20lb in the gym.

Patients 1, 4, and 5 utilized activity modification and physical therapy as treatment modalities. All three had mild improvement of their pain symptoms and had no further loss of ROM. Patients 4 and 5 were prescribed bisphosphonates in addition to their activity modification and physical therapy treatments.

## Discussion

Atraumatic elbow AVN in the adult population is a rare but disabling clinical entity that has not been extensively investigated. The purpose of this study was to evaluate a cohort of adult patients with atraumatic elbow AVN, specifically the anatomic distributions of AVN within their elbows and the risk factors for its development. The findings from our study include the following: the most frequently affected locations of the elbow were the capitellum and trochlea, but commonly there was involvement of the proximal radius and/or proximal ulna as well; all patients had a pre-existing diagnosis of AVN of a different joint prior to elbow AVN diagnosis; and all patients had taken high-dose oral corticosteroids.

In the hip and the shoulder, two of the most commonly affected joints by atraumatic adult AVN, one bone is exclusively involved (the femoral head and the humeral head, respectively).^5^^,^^10^ We hypothesized, based on our clinical experience, that this would not be the case for the elbow, which would instead demonstrate a variety of affected locations. We found this to be true — most patients in our study had portions of the distal humerus epiphysis, including the capitellum (88%) and the trochlea (88%), affected, but the proximal radius (44%), the proximal ulna, and olecranon (33%) were frequently involved as well. This is likely secondary to the watershed areas of the anterior distal humerus. Thus, the capitellum, trochlea, and olecranon are at increased risk for loss of blood supply.^7^^,^^13^ The radial collateral artery, which branches from the deep brachial artery, supplies the lateral distal humerus and anastomoses with the radial artery distally. The superior and inferior collateral arteries supply the medial aspect of the distal humerus and anastomose to the anterior and posterior recurrent artery before joining with the ulnar artery distally.^13^ Although the pathology of AVN is not entirely understood, the increased risk of loss of blood supply due to anatomic vascularization patterns would increase the risk of these sites being affected. This varied distribution is also true with secondary osteonecrosis of the knee, in which a reported 20% of cases involve the proximal tibia instead of or in addition to the more commonly affected distal femur. It may be that the similar development and bloody supply of the knee and elbow make these joints more likely to be affected by multifocal AVN.

The optimal treatment for adult elbow AVN is in the very early stages of discovery. To our knowledge, there have been no medical studies describing surgical treatment options available for this condition. In our opinion, based on the clinical success of core decompression surgery in the shoulder^10^ and hip,^4^ it may be that core decompression with or without elbow arthroscopy is a reasonable procedure for patients with precollapse elbow AVN who have failed conservative treatment. The early results from this procedure performed in two patients in this cohort are promising, but of course much too early to elicit any conclusions. It remains to be seen what the optimal surgical treatment for this condition is, and whether and how other sites of AVN (eg, involved proximal ulna and radius) should be treated. Of the two patients who underwent operative intervention, one had full subchondral collapse and joint space narrowing while the other was precollapse. The goal of operative intervention for patient 2 with stage V osteonecrosis was to return ROM as well as pain relief. For Patient 6 with stage II osteonecrosis, goals for intervention were to avoid any subchondral collapse and preserve the joint space. Both patients have reported an improvement in pain at their most recent follow-up (9 and 8 months, respectively).

We found that all patients with elbow AVN in our study had previously been diagnosed with AVN of other joints prior to developing elbow symptoms. This is consistent with the relative rarity of elbow AVN as opposed to other joints. It is interesting that the elbow is relatively “protected” compared to these other joints; one possibility is the dense blood supply that the elbow enjoys is protective. This finding that the elbow was clinically affected only after at least one other joint in this population will hopefully encourage clinicians evaluating adult patients with isolated, atraumatic elbow AVN to investigate other more commonly affected joints.

Prolonged systemic high-dose corticosteroid use is thought to cause AVN due to osteocyte apoptosis, which subsequently disrupts the lacunar-canicular system.^10^ There has not been a documented correlation in dosage and duration of corticosteroid to development of elbow AVN. Patients who had only 1 year of steroid use had radiographic evidence of collapse. Thus, larger multicenter studies will be required to elucidate or better characterize this relationship.

Autoimmune disorders and chemotherapy may also have a role in the development of AVN. Patients with SLE had the highest risk of AVN compared to other autoimmune disorders.^11^^,^^12^ There are limited case reports describing the development of AVN in patients undergoing chemotherapy. Harper et al described that bleomycin and vinblastine are the most likely to causes AVN.^3^ The pathophysiology is thought to be secondary to production of cytokines from chemotherapy medication leading to formation of thrombi and arterial blood supply disruption leading to AVN.^4^ Among our cohort, one patient had SLE, another dermatomyositis, while the rest had cancer and received chemotherapy in addition to steroids. However, further studies are needed to analyze and account for any confounding factors.

Limitations of this study include possible underreporting due to study design limitations, the small cohort number, and a lack of patient-reported outcomes. The initial patient “filter” relied on the patient having received an MRI and the report to have included the keywords “necrosis”, “avascular” and/or “AVN”. If the patient was not able to undergo MRI for any reason, or these keywords were not used by the radiologist, the patient would have been falsely excluded. While the cohort presented in this study is relatively small, it is the largest population of adults with atraumatic elbow AVN presented to our knowledge; larger studies are preferable and will likely require multicenter collaborations. Our study lacks patient-reported outcomes as their use was too infrequent amongst providers to warrant inclusion.

## Conclusion

Atraumatic AVN of the adult elbow is a rare entity. When present, it is more likely to be multifocal than other, more commonly affected joints. Patients typically have other joints affected prior to elbow AVN diagnosis. And high-dose systemic corticosteroid use, whether initiated for oncologic or rheumatologic treatments, appear to be highly correlated to the development of elbow AVN. Larger, collaborative studies are required to expound upon these findings. The eventual development of surgical treatment options should be based upon the understanding of elbow AVN development, anatomic location, risk factors, and natural history.

## Disclaimers

Funding: This study was funded by 10.13039/100000002NIH T32 grant.

Conflicts of interest: Andrew Jensen is a board or committee member of AAOS and a paid consultant for FH Ortho and Zimmer. All the other authors, their immediate families, and any research foundation with which they are affiliated have not received any financial payments or other benefits from any commercial entity related to the subject of this article.
